# Opportunistic automated aortic valve calcification assessment in low-cost, screening CT calcium score exams

**DOI:** 10.21203/rs.3.rs-6630820/v1

**Published:** 2025-06-02

**Authors:** Ananya Subramaniam, Hao Wu, Sepideh Azarianpour, Naomi Joseph, Tao Hu, Neda Shafiabadi Hassani, Ammar Hoori, Sanjay Rajagopalan, Sadeer Al-Kindi, David L. Wilson

**Affiliations:** 1 Department of Biomedical Engineering, Case Western Reserve University, Cleveland, OH, 44106, USA; 2 Harrington Heart and Vascular Institute, University Hospitals Cleveland Medical Center, Cleveland, OH, 44106, USA; 3 Center for Computational and Precision Health (C3PH), DeBakey Heart and Vascular Center, Houston Methodist, Houston, TX 77030, USA; 4 Department of Radiology, Case Western Reserve University, Cleveland, OH, 44106, USA

**Keywords:** Aortic valve stenosis, aortic valve calcification, CT calcium score, parameter regression

## Abstract

Aortic valve stenosis (AS) is the most common valvular disease, with a growing impact in the aging population. AS can culminate in heart failure if left untreated. Early treatment with minimally-invasive transcatheter aortic valve replacement (TAVR) is being evaluated in clinical trials. We addressed an unmet clinical need for early detection, referral to echocardiography for AS evaluation, and possible treatments (e.g., lifestyle changes, drugs, or TAVR). As aortic valve calcification (AVC) is typically present in AS, we created a method to detect AVC in low-cost/no-cost non-contrast CT calcium score (CTCS) screening exam images. We developed a multi-task deep network to identify a cylindrical aortic valve region of interest (ROI) and applied Agatston criteria within the ROI to obtain calcifications. Predicted ROIs had good agreement with cardiologists’ labels and sometimes were better. Predicted Agatston scores agreed with cardiologists (r=1.00, paired t-test p=0.573, t=0.57). On a retrospective screening cohort of 2000+ patients, we found that 20.6% of men and 22.2% of women had some degree of AVC. According to guidelines, 3.53% and 9.04%, respectively, would have severe AS. These promising results indicate that further evaluation of this approach is warranted, with the potential for significant public health impact.

## INTRODUCTION

Aortic valve disease is the most common form of valvular disease, with growing prevalence due to increased population aging ^1-3^. The most prevalent form of aortic valve disease is calcific aortic valve stenosis (AS), characterized by progressive calcification of the valve leaflets ^4^. Without diagnosis and intervention, patients with severe AS face poor prognosis as the condition can lead to heart failure and has an estimated 5-year survival rate of only 15-50% ^5^. In recent years, transcatheter aortic valve replacement (TAVR) has emerged as a highly successful valve replacement procedure that is less invasive and lower risk than its traditional surgical alternative ^6^. Recent studies have explored the benefits of early TAVR in patients with asymptomatic severe aortic stenosis, suggesting that early AS detection and TAVR intervention can be beneficial, ultimately reducing morbidity and mortality from this condition ^7^. The gold standard for AS assessment, diagnosis, and severity grading is echocardiography, specifically transthoracic echocardiography (TTE) ^8^. Patients are referred for echocardiographic evaluation when they exhibit risk factors such as advanced age, bicuspid aortic valve, or family history of valvular disease, or present with symptoms including abnormal heart sounds, shortness of breath, chest pain, angina, syncope, or abnormal ECG ^9,10^.

Non-contrast CT scans and low-cost/no-cost CT calcium score (CTCS) exams performed for risk assessment may allow opportunistic screening for AS through analysis of aortic valve calcifications (AVC), allowing for early echocardiography referral based on the presence of valve calcification. If integrated into the current clinical standard-of-care, detection of AVC in a CTCS exam could alert clinicians to the need for surveillance and possible referral to echocardiography. There is a potential to turn routine CTCS exams into a critical opportunity to identify patients with disease early who might otherwise be missed until damage is done. In addition, our approach could help identify patients suitable for early TAVR or drug treatment. Early detection will be essential if any of the five emerging drug treatments are accepted for reducing elevated lipoprotein(a) levels as a treatment for AS ^11^. AS is currently diagnosed using hemodynamic measurements from echocardiography, including aortic valve area, trans-valvular pressure mean gradient, and peak aortic jet velocity ^12^ or similar measurements from MRI. However, in patients with low-flow low-gradient or paradoxical low-gradient AS, these echocardiography parameters are not reflective of AS severity ^10^. In such cases, aortic valve calcium score from CTCS images serves as an informative complementary diagnostic modality^13^.

There is a need for robust automated analysis of AVC in non-contrast CTCS images, both to enable earlier detection of AS for timely intervention via TAVR and to aid in diagnosing low-flow, low-gradient cases where echocardiography measurements may be insufficient. Comparable work aiming to identify AVC in non-contrast images is limited. Most work on aortic valve segmentation has been done on contrast CT images for preprocedural TAVR planning ^14-18^. Much less has been done in non-contrast images on automated analysis of the aortic valve and its calcifications. Chang et al. developed a model to automatically segment individual AVC from non-contrast CT images with promising results, training on manually-labeled AVC segmentations ^19^. Sensitivity was good, but they reported false positives from misidentification of calcification from other heart regions. Another approach created patient-specific aorta and aortic valve segmentations in non-contrast images using registered paired CTA images as a guide ^16,20^. Briefly, non-contrast and CTA images were registered, allowing labels in CTA images to be copied to the non-contrast images. Non-contrast images and labels were then used in nn-UNet to develop a deep learning segmentation model. The authors did not report on AVC detection. Identification of coronary calcifications in non-contrast images is very well studied with excellent results ^21-24^, suggesting success with AVC detection. There remains an unmet need for a robust, spatially localized method for automated identification and analysis of AVC in non-contrast CT images. Importantly, AVC has been exclusively analyzed using the AV-Agatston score, which sums up calcified voxels in the aortic valve region without consideration of spatial distribution.

We have developed novel multi-task deep learning methods for identification and analysis of AVC in non-contrast CT calcium score exams using automated detection of a cylindrical aortic valve region of interest (ROI) and application of Agatston criteria for calcification segmentation within the region. We validated our methods for automated AVC identification using manually annotated ground truths, and evaluated our methods in a large external screening cohort (2000+ cases) to assess the incidence of AVC and demonstrate the importance of incorporating AVC analysis in CTCS screening images. Our method includes the deep learning identification of a unique geometrical model that enables calcification localization, offering a promising solution for automated AVC identification and analysis.

## METHODS

### Overview

**Figure 1.**
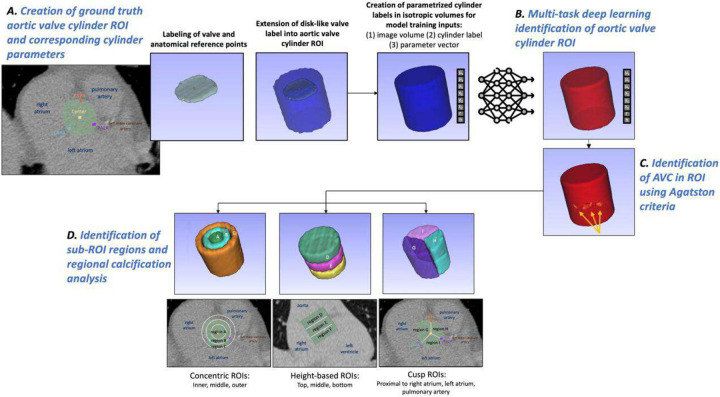
Image analysis workflow. A. Manual labeling of the valve in a multiplanar reformatted CTCS image volume aligned with the valve plane (green), and auxiliary points placed based on the anatomical references of the pulmonary artery, left atrium, and right atrium (named center, PALA, LARA, RAPA). This was followed by the extension of disk-like annotation to cylinder ROI using PCA, and cylinder parametrization to create a perfect cylinder in 3D space parametrized by 8 values (normal vector in x, y, z, origin (x, y, z), radius, and height). B. Deep learning cylinder segmentation using our three-branch learning scheme, trained to automatically generate cylinder parameters of the aortic valve ROI from an incoming CTCS image. The automated masks were used to identify aortic valve calcifications using Agatston criteria (voxels ≥ 130 HU with a 2D area≥ 1mm^2^). C. Identification of sub-ROI regions for regional calcification distribution analysis and calcification assessment within them using aortic valve calcification masks.

We developed an image analysis pipeline to automatically detect AVC from CTCS images by identifying a cylindrical aortic valve ROI using multi-task deep learning and applying Agatston HU and size criteria to obtain calcifications (Figure 1). To identify “ground truth” aortic valve cylinder ROIs, cardiologists manually labeled the aortic valve in a single multiplanar reformatted slice. We extended this disk-like valve annotation into a cylinder. We then made our images and cylinder labels isotropic and determined a parameterized, idealized cylinder to create our ground truth inputs for multi-task deep learning (cylinder ROI labels and corresponding eight parameters to define the cylinder in acquisition space) (Figure 1A). At inference, the deep learning approach takes an input CTCS image and outputs the geometric parameters defining a cylinder, which can be reconstructed to create our aortic valve ROI mask (Figure 1B). Within this mask, we detected AVC and calculated features (e.g. AV-Agatston, mass, and volume scores, and the number of lesions) (Figure 1C). Within an ROI, we identified sub-ROIs, allowing us to determine the spatial distribution of calcifications to discriminate between leaflets, wall, and root locations (Figure 1D). The cylinder ROI model enables regional calcification analysis and flexible refinement of the region by adjustment of predicted parameters. After evaluation of training results, the method was applied to evaluate AVC on a separate screening cohort of 2196 CTCS images, demonstrating the utility of such an automated method in detecting patients at risk of AS.

### Images and creation of training data

We used a training dataset of 310 non-contrast coned-down CTCS images – 202 aortic stenosis negative (ASN) and 108 aortic stenosis positive (ASP) – with AS diagnoses confirmed by echocardiography. Of the ASP patients, the cohort contained 62 cases with mild AS, 32 with moderate AS, and 14 with severe AS, 5 of whom underwent TAVR (Table 1). Our cohort comes from a de-identified CTCS dataset (UH CLARIFY) as approved by the Institutional Review Board (IRB) of the University Hospitals Cleveland Medical Center. Images were acquired using a range of CT scanners using 120-kVp and 30-mAs. The “coned down” images ranged from 0.33-0.59-mm in-plane voxel spacing, had either 2.5mm or 3mm slice thickness, and were reconstructed using either a B or CB Philips kernel. All images had an original size of 512x512 in the x−y plane and ranged from 39 to 80 slices.

### Noise management and image curation

As CTCS images can be noisy, we found it necessary to introduce noise reduction and data cleaning on our retrospective images. CTCS images may be subject to noise if an improper reconstruction kernel is used or if x-ray exposure is low or increased insufficiently to account for body size. In many of the noisier images, there were streak artifacts due to photon starvation (Supplemental Figure 1). In our cohort of “coned down” images, the Philips reconstruction kernels used were B (62.3%) and CB (37.7%). Both gave similar noise characteristics and much less noise than some kernels used to reconstruct the entire chest in a manner suitable for lung evaluation (e.g., Philips kernels YA and YB). To assess noise, we segmented the descending aorta using TotalSegmentator and calculated the mean and standard deviation of all voxels < 130-HU (to eliminate any candidate calcifications) within the aorta volume ^25, 26^. Problematic noisy cases had standard deviations > 36-HU. To reduce false positive predicted calcifications in these images, we applied a 2D 3x3 median filter on the noisy images for denoising prior to AVC identification. A median filter is well suited for reducing streaking artifacts. Cases with a standard deviation for voxels under 130-HU > 45-UH were excluded as they were too noisy for automated analysis (Supplemental Figure 1).

We manually created cylindrical ROIs encapsulating the aortic valve region as a step towards providing ground truths for our multi-task deep learning method. As the aortic valve is low contrast and difficult to identify in non-contrast CTCS images, we found it desirable to create multi-planar reformatted image slices in the aortic valve plane for manual labeling. Cardiologist annotators selected the candidate slice corresponding to the emergence of the left main coronary artery and segmented the nearly circular valve area within this slice. In addition, the center point of the valve and three auxiliary points were labeled. Points were placed: 1) between the pulmonary artery and left atrium, 2) between the left atrium and right atrium, and 3) between the right atrium and pulmonary artery (Figure 1). These three auxiliary anchor points allowed us to analyze data with respect to sectors of the cylinder in pie-shaped sub-ROIs.

**Figure 2.**
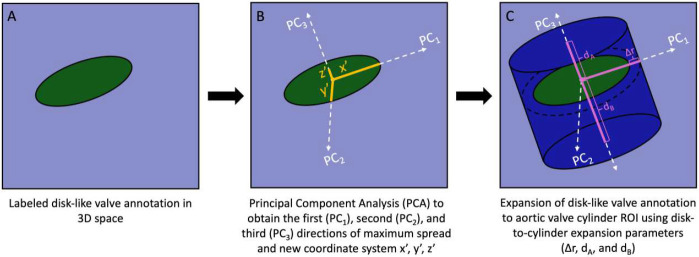
Extension from disk-like valve annotation to cylinder ROI. A. The 3D coordinates of every voxel in the disk-like valve annotation, visualized here as a disk, was rescaled to work in isotropic space. B. PCA was applied to obtain the first (${\mathrm{PC}}_{1}$), second (${\mathrm{PC}}_{2}$), and third (${\mathrm{PC}}_{3}$) directions of maximum spread, converting each voxel to a new coordinate system, x’, y’, z’. C. The disk-like valve annotation was expanded into the aortic valve cylinder ROI using the disk-to-cylinder expansion parameters (Δr, $d_{A}$, and $d_{B}$), with x’ and y’ dictating expansion in the disk plane (radial expansion) and z’ dictating expansion in along the normal vector of the disk (height expansion).

The disk-like valve annotation was extended to an idealized, parameterized aortic valve cylinder ROI. The 3D coordinates of every voxel in the disk-like valve annotation segmentation was rescaled by the voxel spacing to work in isotropic space (Figure 2A). Principal component analysis was applied to those disk voxels to obtain the first (${\mathrm{PC}}_{1}$), second (${\mathrm{PC}}_{2}$), and third (${\mathrm{PC}}_{3}$) directional vectors of maximum variance. As a result, each voxel was in the new coordinate system, x’, y’, and z’ with respect to the labeled disk plane (Figure 2B). x’ and y’ defined a point’s in-plane radial displacement, so these coordinates defined the disk plane. z’ spanned the distance along the disk’s normal vector up and down the aortic valve. Valve radius was identified from the disk-like valve annotation as the largest in-plane distance from the disk-like valve’s center to the furthest point of the boundary of the annotated disk. Voxels were assigned to the cylinder using our final radius (valve radius + Δr), distance above the centroid of the valve ($d_{A}$), and distance below the centroid ($d_{B}$) based on radial distance and height distance using Equations (1) and (2). This extension of the cylinder ROI was conducted on the original, non-isotropic volumes while taking voxel size into account, with measurements computed in millimeters. Disk-to-cylinder expansion parameters (Δr, $d_{A}$, and $d_{B}$) were empirically optimized as described next (Figure 2C). Expanded cylinder masks were saved in the original (non-PCA, non-isotropic) coordinate space.

Cardiologist annotators labeled AVC in the cohort to enable cylinder dimension optimization and provide a manual AVC ground truth. In addition, we had clinical labels of coronary artery calcifications, previously labeled in a semi-automated manner by clinical experts. Disk-to-cylinder expansion parameters (Δr, $d_{A}$, and $d_{B}$) were manually optimized to include AVC while excluding coronary calcifications. Values of Δr, $d_{A}$, and $d_{B}$ were selected to be 2-mm, 10-mm, and 30-mm, respectively. The radial addition of 2mm helped account for the variation in the width of the valve. The height of the cylinder (40-mm) was within the range of the heights of replacement valves used for TAVR, effectively being large enough to capture variations in heart size ^27^. Cardiologists reviewed final cylinder masks to confirm validity of the ROIs.

We created isotropic volumes of a standard size for our deep learning method by resizing images and cylinder masks to isotropic voxels (1-mm on a side). To standardize isotropic image sizes for model training, the boundaries of the heart sac, obtained using an existing model developed by our group ^28^, were used to crop all volumes to 176x176x128 isotropic voxels. Cropping coordinates were stored for future reference, as original (non-isotropic) images were used for calcification metric calculation downstream.

We created our final dataset for model training, consisting of isotropic images, cylinder masks, and cylinder parameter vectors. As a result of all previous steps, each aortic valve cylinder ROI mask could be fully described by eight geometric cylinder parameters: three parameters corresponding to the normal vector ${\mathrm{PC}}_{3}\;(\mu_{x},\mu_{y},\mu_{z})$, three parameters for the cylinder origin obtained from the center of mass of the cylinder ($x_{0}$, $y_{0}$, $z_{0}$), the radius r (radial displacement + Δr), and the height $h\;(d_{A}+d_{B})$. These cylinder parameter values were used to reconstruct each cylinder label in a clean, parameterized fashion, such that there was a direct mapping between parameter values and the isotropic cylinder label. This was required for our multi-task deep learning structure, which compared predicted parameters with ground truth parameters as well as reconstructed masks from predicted parameters to ground truth masks.

### Deep learning algorithm and evaluation

#### Algorithm

**Figure 3.**
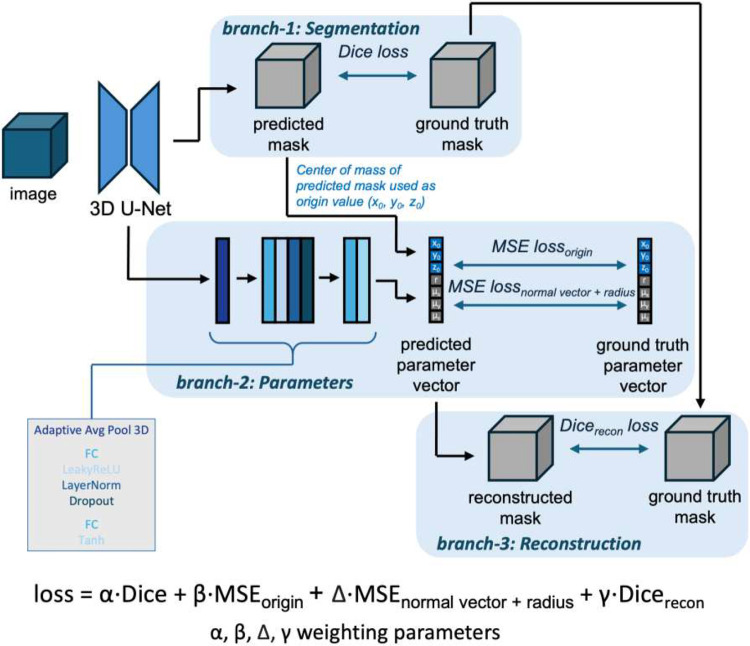
Multi-task deep learning for automated generation of AV cylindrical ROI. Inputs are isotropic images and labels, and seven cylinder parameters. Images are fed into a 3D U-Net backbone, which has both a semantic segmentation output (branch-1), validated against the ground truth cylinder mask, and a latent space output that is fed through an MLP for parameter prediction (branch-2), validated against the ground truth parameter vector. The predicted parameters are used to reconstruct a cylinder mask (branch-3), which is validated against the ground truth cylinder mask. From the predicted ROI segmentation (branch-1), we calculate the center of mass to estimate the cylinder origin, a step that improves results as compared to direct deep learning estimation of all parameters. The final loss is a combination of the Dice loss from branch-1, the two MSE losses from branch-2, and the Dice_recon_ loss from branch-3.

We created a multi-task deep learning approach to automate the extraction of aortic valve cylindrical ROI parameters from a CTCS image (Figure 2). Our multi-task method was designed to leverage the spatial context of the cylinder segmentation to improve the regression of cylinder parameter values. We therefore designed the task with three branches, based on a 3D U-Net backbone (Figure 3). Branch-1 consisted of the semantic segmentation task, learned using a Dice loss between the predicted cylinder segmentation and label. Branch-2 (parameter values) and branch-3 (reconstructed segmentation) operated jointly to refine the overall output. Branch-2 pulled from the latent space output of the U-Net encoder (shape 256x11x11x8) and passed this output through an AdaptiveAveragePool3D layer to reduce the large dimensions to 256x1x1x1 while maintaining the unique spatial representation present in the latent space output. This output was then passed through a regularized MLP block (FullyConnected, LeakyReLU activation, LayerNorm, Dropout), followed by a FullyConnected layer and tanh activation, resulting in an output of size *batch_size* x 7. We regressed to seven parameters for the cylinder (the height h was 40-mm ($d_{A}+d_{B}$) for all cases). These predicted parameters were compared against the ground truth cylinder parameters using Mean Squared Error (MSE) loss. The predicted parameters were used to reconstruct a cylinder mask (branch-3), which was validated against the ground truth label mask using Dice loss (termed Dice_recon_). The total backpropagated loss was a combination of the losses from branches 1, 2, and 3. The predicted ROI segmentation was used to calculate the center of mass, which became the predicted cylinder origin (Figure 3). This step leveraged the spatial context learned during the semantic segmentation task (branch-1) and reduced the number of parameters to regress, improving results as compared to deep learning regression of all parameters. All other cylinder parameters were obtained via deep learning regression in branch-2, finally giving all seven parameters ($\mu_{x}$, $\mu_{y}$, $\mu_{z}$, $x_{0}$, $y_{0}$, $z_{0}$, r). The multi-task loss in Figure 3 was a combination of Dice loss (branch-1), MSE_origin_ (branch-2), MSE_normal vector + radius_ (branch-2), and Dice_recon_ loss (branch-3), with weighting factors α, β, Δ, γ=1∕4. A batch size of 8 was used and the model was trained for 600 epochs using a 48GB GPU.

For model training, inputs were isotropic CTCS image volumes, corresponding isotropic cylinder labels, and the seven ($\mu_{x}$, $\mu_{y}$, $\mu_{z}$, $x_{0}$, $y_{0}$, $z_{0}$, r) cylinder parameters. The eighth parameter, height h for all cylinder ROIs, was fixed at 40-mm. Normal vector parameters inherently ranged between −1 and 1, and the radius parameter, originally ranging between 16 and 22mm, was rescaled to range [−1, 1] to maintain scale consistency when applying tanh activation using $r_{rescaled}=2\left(\frac{r}{40}\right)-1$. Data was divided into 65% training, 15% validation, and 20% held-out test, giving 200 cases reserved for training, 49 for validation, and 61 for testing. The PyTorch and MONAI libraries were used for model development. All images were adjusted using a window of [−300-HU to 100-HU], and rescaled between 0 and 1. The addition of random Gaussian noise (mean=0, std=0.1) was used for mild intensity data augmentation during training. Displacement augmentation was laborious as it had to be conducted via offline augmentation (with corresponding change in origin parameters). This was used during one network training experiment, but was abandoned as it greatly increased training time with negligible impact on results.

### AVC evaluation and analysis

We evaluated the ability of our algorithm to identify manually annotated AVC. From predicted parameters, we generated predicted ROI cylinders within the original image acquisitions, accounting for cropping and rescaling. Within the predicted ROI cylinders, the Agatston criteria for coronary artery calcium evaluation was employed − ≥ 1-mm^2^ connected voxels ≥ 130-HU in a 2D slice (i.e. 7 connected voxels for a voxel size of [0.4-mm, 0.4-mm]) ^29^. The Agatston score is calculated as a weighted average of calcium voxels, incorporating calcification density as determined by max HU value in the lesion. The volume score is obtained by summing the number of voxels and accounting for voxel size, and the calcification mass score is calculated using a calibration factor ^30^. To increase accuracy for the number of lesions metric, it was calculated as the number of connected components (lesions), with a valid lesion requiring an Agatston score > 10 Ag, ensuring noise was not erroneously included in the lesion count. The coronary Agatston score for the cohort was calculated from cardiologist-annotated masks. We used the same standard coronary calcium measurements to calculate the aortic valve calcification scores, which have been used in the literature to quantify AS severity.

Using the ground truth cylinders, regional calcification metrics were evaluated by dividing the cylinder into sub-ROIs to analyze distribution. The sub-ROIs were generated to provide spatial context to calcifications: concentric sub-ROIs demonstrate whether calcifications tended to adhere to the aortic wall or were central, height-based sub-ROIs assess whether calcifications were present closer to the aortic root or aortic leaflets, and cusp sub-ROIs indicate whether calcifications fall proximal to the right atrium, left atrium, or pulmonary artery (Figure 2D). The cusp sub-ROIs were divided based on the auxiliary points assigned during labeling, and give anatomical context rather than explicit leaflet divisions, as it is difficult to visualize leaflets in non-contrast images. The height sub-ROIs were generated using fractional heights of the full ROI, and the concentric sub-ROIs were created by generating cylinders of fractional radii. We additionally further separated these 9 sub-ROIs to generate 27 sub-ROIs within which the Agatston scores were calculated and heatmaps were generated.

## RESULTS

### Aortic valve calcification assessment

**Figure 4.**
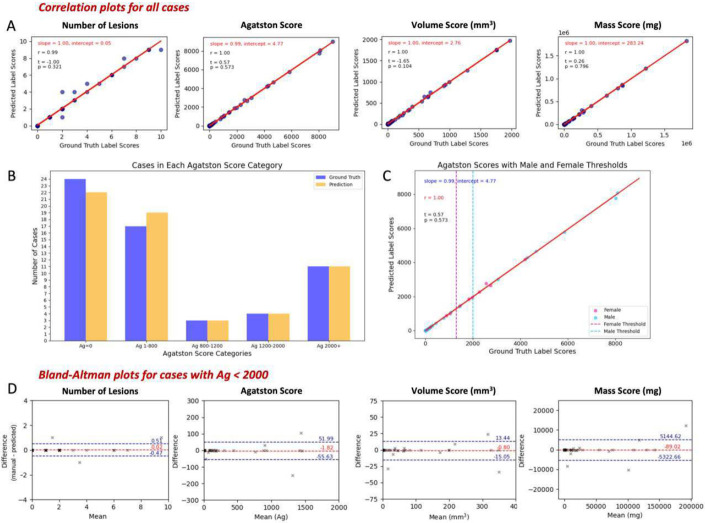
Comparison of automated AVC assessments to manual ground truth. Over the held-out test set (N=58), scatter plots of automated number of lesions, Agatston, volume, and mass scores show excellent correlation with results from ground truth annotations. A. Slopes, intercepts, and Pearson correlation coefficients all indicate near ideal results. B. There is excellent agreement for AV Agatston categories (test cohort, N=58). The only 2 misclassified cases had Ag=0 (annotation) and clinically inconsequential Ag~10 (automated). C. A scatter plot of the test cohort with vertical lines for male and female severe AS thresholds again shows excellent correlation and participants above thresholds. D. Bland-Altman plots for Ag<2000 (to aid visualization) show little bias and spread (N=48).

We assessed AVC segmented by our automated method versus cardiologist-annotated ground truth results in the 61 held-out test cases, from which the three noisiest cases were excluded. Scatter plots in Figure 4A show excellent agreement with the manual results for the numbers of lesions, Agatston, mass, and volume. The slopes of the regression lines ranged from 0.99-1.00, and the intercepts were near zero, supporting strong agreement with the ground truth. Pearson correlation coefficients (r) ranged from 0.99-1.00. Additionally, the results of paired t-tests for all metrics showed no significant bias (p > 0.05).

Agatston scores for the cohort were reported in bins relevant to clinical outcome for AVC (Figure 4B) with bins determined with the following considerations. The European Society of Cardiology Guidelines state that severe AS likely corresponds to an AV-Agatston score > 2000 in males >1200 in females ^31^. Guidelines additionally state that severe AS is considered unlikely with an AV-Agatston score < 1600 in men and < 800 in women. We selected bins of Ag=0, 1-800, 800-1200, 1200-2000, and 2000+. There were only two cases of reclassification as compared to manual analysis. In both instances, manual analysis gave Ag=0 and predicted gave Agatston score > 0 (low Agatston scores of values of 11.44 and 9.41, likely inconsequential differences). Agatston scores were plotted over the full range of values with literature cutoffs indicated for severe AS in men and women (Figure 4C). Multiple participants were above thresholds, and men tended to have the highest scores in our held-out test set. As any patient with an Agatston score > 2000 would be considered at risk of severe AS, with immediate referral to echocardiography for further evaluation, we chose to plot data with ground truth Ag < 2000, giving 48 out of 58 test cases in Bland-Altman plots (Figure 4D). All Bland-Altman plots gave reasonable mean bias and 95% limits of agreement (LoA) for the number of lesions, Agatston score, volume score, and mass score. Agatston score had a very low mean bias of −1.82 (Figure 4D). We examined outliers in more detail. With respect to Agatston score, there were only 2 cases with differences greater than 100. For both, their manual and predicted scores exceeded an Agatston score of 1200, putting them in a range where an echocardiogram would be recommended regardless of this difference. The maximum difference in volume score was 30-mm^3^, for which there were 2 cases. The bias for each metric was negative, indicating that our method, if anything, slightly overestimates compared to manually-annotated calcifications.

### Assessment of cylinder alignment

**Figure 5.**
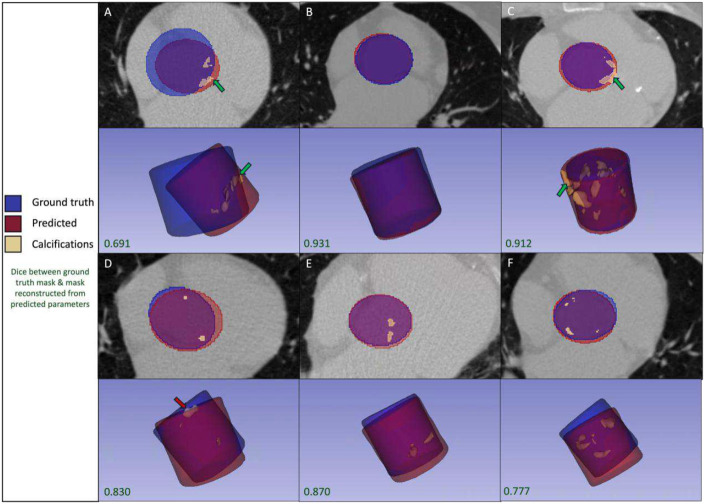
Qualitative results: predicted and ground truth cylinder masks. Visualization of ground truth and predicted (branch 2+3) cylinders and associated calcification masks obtained from each, with Dice scores between ground truth and predicted cylinder masks reported. A. Case with low cylinder alignment where predicted mask captures aortic valve calcifications missed by ground truth cylinder. B. Case with high alignment of ground truth and predicted cylinder masks, no calcification present. C. Case with high cylinder alignment where predicted cylinder mask captures aortic valve calcifications missed by ground truth cylinder. D. Case with medium cylinder alignment where predicted cylinder mask misses a portion of an aortic valve calcification captured by ground truth cylinder. E. Case with medium cylinder alignment where both cylinders capture aortic valve calcifications accurately. F. Case with low cylinder alignment where both cylinders capture aortic valve calcifications accurately.

Ground truth and predicted cylinders were visualized along with associated calcifications (Figure 5). Due to inconsistencies in manual disk-like valve annotation that may have resulted in variations in cylinder size and location, there were cases where our model’s predicted reconstructed cylinder captures true AVC missed by the ground truth cylinder (Figure 5 A, C). The best performing predicted cylinders with Dice scores upwards of 0.9 show extremely high alignment with the ground truth cylinders (Figure 5 B, D, E).

### Ablation study

We conducted ablation studies to evaluate the contribution of individual model components to overall performance. As cylinder Dice is not very sensitive for identifying alignment, we added other metrics (i.e., Hausdorff distance, the absolute difference in radius, the Euclidean distance from the origin, and the cosine similarity of the normal vector). An architecture with branch-1 alone gave good segmentation metrics but did not provide cylinder parameters. Parameter regression alone (branch-2 and branch-3) gave inferior results. Adding branch-1 (3^rd^ row) gave little improvement, despite making adjustments to architecture, parameter rescaling, and MSE loss calculation, the origin values were not sufficiently accurate, preventing Dice_recon_ in the held-out test set from exceeding 0.625 without linkage between branches 1 and 2+3. Only when we included the center of mass from the segmentation (4^th^ row) to identify the cylinder origin did we get superior results. Evidently, deep learning was challenged to identify all seven cylinder parameters due to interactions of terms. This was obtained even with a number of training variations.

### Regional aortic calcification analysis

**Figure 6.**
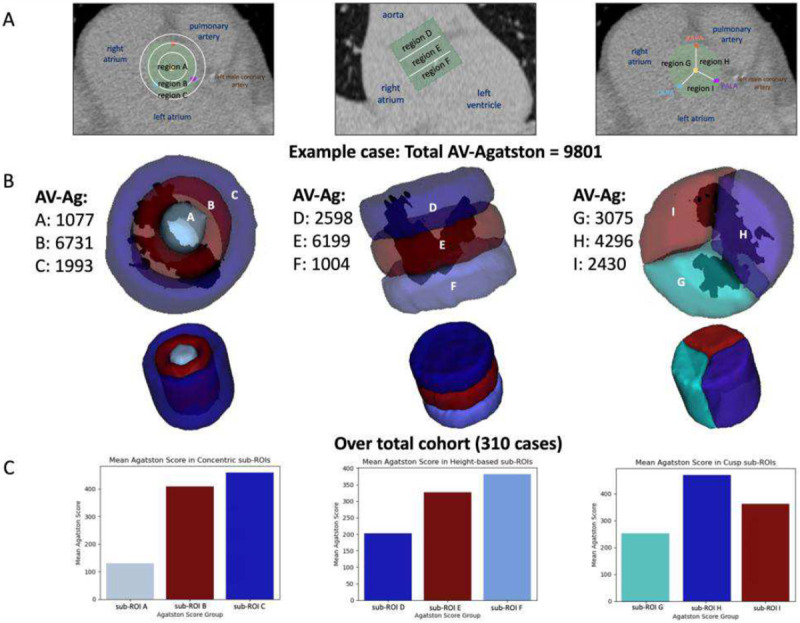
Cylindrical ROI sub-region analysis. A. Nine sub-regions (A-I) are identified with appropriate overlays on a CTCS image volume in. B. Dark regions within the transparent cylinder views correspond to calcifications for a single participant. This participant has a very high Agatston score with heavy calcification in the A and B central regions. C. We create average sub-ROI results over 310 cases. Across the three graphs, the outer ring (C) has the highest score; the bottom disk (F) has the highest score; and the rightmost sector (H) has the highest average score.

Calcifications within cylindrical ROI sub-regions were analyzed in order to understand anatomical correlates of calcification accumulation. In Figure 6B, a participant’s aortic valve with heavy calcification was analyzed with the cylindrical ROI divided into nine sub-regions. Dark regions correspond to segmented calcifications. Agatston score within each sub-region was determined. There were heavy calcification concentrations in the H and G regions, suggesting calcification at the boundary of the left and right coronary cusps. Average results across the training cohort of 310 cases is shown in Figure 6C. The mean Agatston score increases when moving from the first to third concentric sub-ROI (A<B<C). The mean Agatston score increased when moving from the first to third height-based sub-ROI (D<E<F). The cusp sub-ROI adjacent to the right atrium had the lowest Agatston score, followed by that adjacent to the left atrium, followed by that adjacent to the pulmonary artery (G<I<H). This corresponds to the boundaries between the non-coronary cusp (NCC) and right coronary cusp (RCC) (G), NCC and left coronary cusp (LCC) (I), followed by LCC and RCC (H).

**Figure 7.**
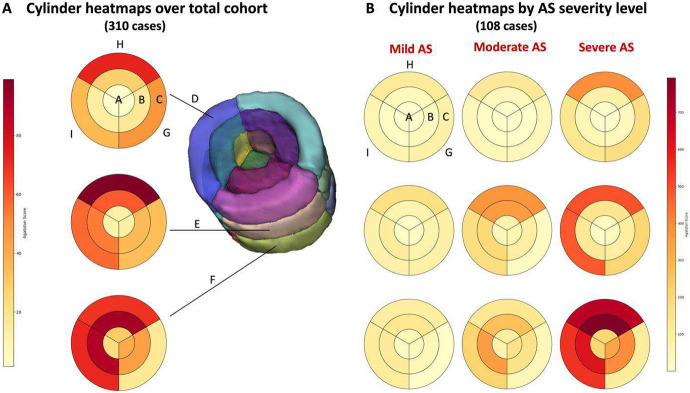
Agatston score heatmaps within 27 sub-ROIs. The full 310-participant cohort is analyzed, and color coding refers to the average Agatston score within a sub-region. A. Heatmaps over the entire cohort, with three height-based ROI slabs (D-F) that are also visible in the 3D cylinder rendering. B. AS positive cases are analyzed stratified by AS severity (mild, moderate, and severe). Relatively little calcification is seen in the top row (D) region even for severe AS as compared to the bottom (F) region which corresponds to the valve root vs. valve cusps. Also remarkable in the bottom row is the accumulation of calcification in regions B-H and C-H. The left coronary cusp will lie between sectors H and I, which both have a high accumulation of calcification. This data suggests that this leaflet might be involved in severe AS.

We further analyzed spatial distribution by dividing cylindrical ROIs to create 27 non-overlapping sub-ROIs and analyzed results for participants with mild, moderate, and severe AS (Figure 7). On the left, heatmaps of Agatston score were generated over the total training cohort that show calcification accumulation in the bottom slab in regions F-H and F-I, as well as in the middle slab in region E-H-C (Figure 7A). Heatmaps stratified by AS severity for ASP cases provide a picture into the regional accumulation of calcifications and their distribution, with heavier wall and cusp calcification particularly appearing in slabs E and F at H-B, H-C, I-B, and I-C (Figure 7B). These regions of very high calcification in those with severe AS can be associated with the boundaries between the LCC and RCC (H) and the NCC and LCC (I), primarily along the wall (C) but extending starting to extend towards the center of the cusp (B).

### Screening cohort analysis

**Figure 8.**
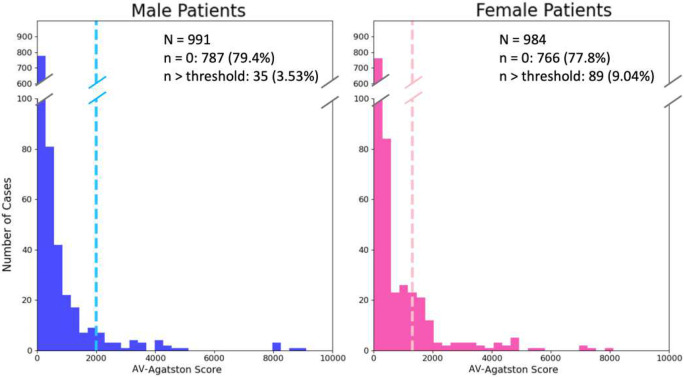
CTCS screening cohort gives a broad distribution of AV Agatston scores for both male and female participants. 79.4% of men and 77.8% of women had Ag=0, and 35 men (3.53%) and 89 women (9.04%) had Agatston scores above the guideline suggested thresholds of 2000 and 1200 Ag, respectively. If we use more conservative thresholds of 1500 and 900, we get 51 men (5.15%) and 114 women (11.6%). Results suggest that many patients in our screening cohort could likely benefit from echocardiography evaluation. In this analysis, 221/2195 cases were deemed unsuitable for automated analysis due to noise in the aorta exceeding our acceptance criteria.

Our automated analysis was applied to the screening cohort of 2196 participants in the CLARIFY registry. Figure 8 shows the distribution of Agatston scores over the cohort for male and female participants. Studies have shown that there are clinically different aortic valve Agatston thresholds in males and females, with women presenting with severe stenosis at lower levels of aortic valve calcification, even when body size differences are accounted for ^32^. This is shown to be physiologically associated with greater fibrosis as opposed to calcium deposition in the AS disease process for females ^13,33^. As noted, the literature states that severe AS is likely with an AV-Agatston score > 2000 in males and an AV-Agatston score >1200 in females ^31^. In our screening population of 2196 patients, 221 cases were excluded due to excessive noise interfering with accurate automated AV-Agaston score calculation. Of the remaining 1975 patients, 35 male patients were above the male threshold of 2000 Ag, with a prevalence of severe AS in men of 3.53%. There were 89 female patients above the female threshold of 1200 Ag, reflecting a prevalence of severe aortic stenosis in women of 9.04%. Over the entire cohort, the prevalence of severe aortic stenosis was 6.28% (Figure 8).

## Discussion

We have demonstrated that our multi-task deep learning network successfully generates cylindrical ROIs encapsulating the aortic valve region, enabling accurate extraction of aortic valve calcifications that align with manual annotations. With training on only 200 cases, we have developed considerable confidence in our predicted ROI cylinders. Gross errors placing the ROI in the wrong part of the heart were never found. We have even identified cases in test data where predicted cylinders were better than manually obtained cylinders (Figures 6A, 6C). As cylinder alignment is not directly reflective of AVC detection accuracy, model efficacy was ultimately evaluated using alignment between the manually annotated AVC and the AVC obtained from predicted cylinders. Examining the impact of ROI placement on subsequent Agatston score calculation, we found very good agreement with the ground truth annotations (Figure 4).

Any errors in the predicted AV-Agatston score should have relatively little impact. When we analyzed AVC severity bins (Figure 4), we found only two misclassifications. For both, ground truth was Agatston = 0 and predictions were Agatston < 12. These small values are inconsequential given the bin choices described next. Our chosen bins reflect Agatston values that are used for critical aortic stenosis cutoffs in the literature, with < 800 being the cutoff for unlikely severe AS in females, > 1200 being the cutoff for likely severe AS in females, and > 2000 being the cutoff for likely severe AS in males ^31^. Bland-Altman and correlation plots showed high agreement between the manual annotation and predicted AVC masks for critical calcification metrics, demonstrating the reliability of our automated method in identifying AVC. Our method’s mild bias towards overestimation of AVC compared to manually-obtained calcifications would not be a clinical problem when using our automated methods for screening and potential referral to echocardiogram.

The idealized cylinder from parameters was advantageous compared to the semantic segmentation output. We found it pertinent to our downstream tasks to generate perfect cylinders as opposed to generalized cylinder segmentations for three reasons: 1) Semantic segmentation relies on image boundaries to determine edges, and therefore may adhere to incorrect regions, causing bumps and edges that accidentally detect coronary calcifications within/outside our region. 2) A perfect cylinder facilitates clean mathematical divisions into sub-ROIs within which we can regionally analyze AVC. 3) Generating cylinder parameters gives us the freedom to tinker with the radius and height of the cylinder in a smooth manner if the user desires manual revision of the automated segmentation – this is much more difficult to do with a free semantic segmentation shape. In addition, the aortic valve cylindrical ROI approach should allow us to differentiate between calcifications in leaflets and the aortic valve ring. The latter calcifications are thought to be less involved in AS and have been omitted in some studies of AS risk ^34^.

Because we created an oriented cylindrical ROI, we can make interesting observations on the spatial organization of calcifications. Figure 6B shows that we can spatially localize calcifications for a single subject. Sub-regions closely associated with leaflets are quite calcified, suggesting that this would be a cause for concern about AS. Over the training cohort, the mean aortic valve Agatston score decreased from the outermost concentric sub-ROI to the innermost (Figure 6). The outermost sub-ROI likely contains most of the regions corresponding to the aortic wall, but because the valve is not perfectly circular, this sub-ROI might also contain calcifications that fall along the edges of the leaflets adjacent to the aortic wall, a location where calcifications appear in early disease stages ^35^. Very advanced disease causes calcifications in the center of the leaflets, so it follows that concentric sub-ROI A had the lowest mean Agatston score (Figure 6). The center doesn’t appear to comparatively be very calcified, even in severe AS (Figure 7). With respect to the height-based sub-ROIs, the mean Agatston score decreased from the bottom sub-ROI to the top (Figure 6). The bottom (F) corresponds to the leaflet region, where more calcifications are likely to occur, and the top (D) corresponds to the aortic root. For the cusp sub-ROIs, the mean Agatston score was highest in the valve region adjacent to the pulmonary artery, followed by the left atrium, and right atrium. Although our cusp sub-ROI divisions do not directly correspond to anatomical cusps as they are not visible in non-contrast CT, the boundary regions between cusps likely exist in our divisions. Anatomically, regions H and I may contain parts of the LCC and the NCC. One study on 2891 patients undergoing hemodialysis found the most calcification in the NCC and similar frequencies of calcification in the RCC and LCC ^36^. This may align with our results; however, this is a small cohort and there is a degree of ambiguity with respect to the cusp sub-ROIs delineation, so more remains to be seen. Severe disease shows calcification all along the aortic wall, though surprisingly not concentrating on sub-ROI G, which may have anatomical comparison to the boundary between the NCC and RCC. For patients with severe AS, calcification has begun to settle in central regions, (i.e. concentric sub-ROIs B and A), meaning the cusps are more significantly calcified. These heatmaps provide a starting point for large-scale calcification distribution analyses and better understanding of the progression of calcification distribution, further exemplifying the value of our aortic valve cylinder ROI method. This kind of analysis will also likely fine-tune AV-Agatston scoring by identifying high risk features for AS.

We have demonstrated the utility of a new pipeline for automatic quantification of AVC in a screening population. Our methods were employed on a large screening cohort of 1975 images. Based on guideline criteria, 6.3% of participants were likely to have severe AS. At our institution (University Hospitals of Cleveland), CTCS imaging is offered at no cost, creating a current population of >135,000 participants. If we extrapolate our findings to this large population, we anticipate 8478 people with a high likelihood of AS requiring additional echocardiographic screening. Given the high percentage of cases in our screening cohort with significant calcification (e.g., AV-Agatston > 800 = 11%, > 500 = 10%, > 100 = 21%), this could lead to a large number of people who might need to be referred to echocardiography or surveilled. Analysis of AV calcification in CTCS screening exams might become an essential tool for identifying patients who will benefit from intervention (e.g., lifestyle changes, emerging drugs) and standard TAVR and early TAVR treatments.

This work, by establishing a reliable method for AVC extraction, has set the stage for more complex population analysis of AVC distribution and eventual linkage to outcomes. We have developed methods enabling opportunistic screening for AS using CTCS exams and have validated the value of such a monitoring system in a large screening cohort. Additionally, there have been studies showing the value of adding AVC and aortic root calcification to MACE and cardiovascular disease risk prediction models ^37-39^. The regional metrics we generated serve as preliminary aortic valve-omics features, laying the foundation for the development of a robust feature set that can facilitate eventual machine learning prediction of aortic valve stenosis severity and major adverse cardiac events.

## Equations


(Equation 1) $$\sqrt{x^{’2}+y^{’2}}<\text{valve radius}+\Delta r$$

(Equation 2) $$-\text{distance below centroid}\;(d_{B})<z’<\text{distance above centroid}\;(d_{A})$$

## Figures and Tables

**Table 1.** Patient cohort demographics. Demographics for the cohort of 310 patients who received CTCS screening exams and the distribution of their aortic valve stenosis diagnoses. Numbers are reported as mean ± standard deviation (min, max), and p-values are coded based on significance levels (<0.0001 ***, <0.001 **, <0.01 *). Some cases are missing demographic information.

*AS cohort* *demographics*	Full AS cohort (N=310)	Aortic Stenosis Positive (N=108)	Aortic Stenosis Negative (N=202)	P-value
**Age**	62.9 ± 9.2 (38, 82)	66.8 ± 8.43 (38, 82)	60.8 ± 8.91 (39, 81)	<0.0001 ***
**Female**	139 (44.8%)	48 (44.4%)	91 (45.0%)	0.944
**BMI (kg/m^2^)**	30.8 ± 6.8 (17.3, 62.4)	33.7 ± 7.97 (19.3, 62.4)	29.2 ± 5.41 (17.3, 50.1)	<0.0001 ***
**White**	273 (88.1%)	92 (84.8%)	181 (89.6%)	0.487
**Smoker**	94 (30.3%)	34 (34.7%)	60 (29.7%)	0.654
**Diabetes**	54 (17.4%)	34 (29.7%)	20 (9.9%)	<0.0001 ***
**MACE**	26 (8.3%)	5 (5.9%)	21 (10.4%)	0.0594
**Statin**	120 (38.7%)	54 (50%)	66 (32.7%)	0.00188 *
**HDL-cholesterol (mg/dL)**	54.7 ± 15.9 (22, 114)	48.3 ± 13.1 (22, 96)	57.4 ± 16.2 (25, 114)	0.000121 **
**LDL-cholesterol (mg/dL)**	119.7 ± 38.5 (37, 254)	116.9 ± 36.3 (44, 237)	121.0 ± 39.4 (37, 254)	0.492

**Table 2.** Ablation study for model architecture selection. Table of ablation study with respect to label and predicted cylinders, evaluated using mask Dice scores, Hausdorff distances, predicted radius absolute difference, origin Euclidean distance, and normal vector cosine similarity. Note that for the branch 1 only experiment, the output is a semantic segmentation output, and therefore not a clean parametrized cylinder, making the result suboptimal in comparison to clean cylindrical outputs that can be used for calcification distribution analysis and parameter modification. The branch 2+3 experiment involved only the 3D U-Net encoder, whose latent output was fed through the same layers used in our final model to predict parameters, as well as the reconstruction of a cylinder mask from predicted parameters, which was validated against the ground truth mask. The branch 1+2+3 experiment had all branches working concurrently, but failed to improve mask Dice score, largely due to issues in origin identification. Our final proposed model is branch 1+2+3 with use of the center of mass obtained in branch 1 as the origin parameter values, connecting all three branches.

	Predicted Cylinder				
	Mask Dice	Hausdorff Distance	Radius (Absolute Difference in mm)	Origin (Euclidean Distance)	Normal Vector (Cosine Similarity)
**Branch 1 only**	0.815 ± 0.07 **	9.279 ± 3.79	--	--	--
**Branch 2 + 3 only**	0.625 ± 0.16	14.285 ± 7.29	2.046 ± 1.276	11.46 ± 2.27	0.981 ± 0.03
**Branch 1 + 2 + 3**	0.624 ± 0.18	14.292 + 7.32	2.057 ± 1.277	11.54 ± 2.56	0.980 ± 0.03
**Branch 1 + 2 + 3 with CoM to origin linkage – final model**	**0.817 ± 0.07**	**7.218 ± 2.57**	**1.847 ± 1.28**	**2.19 ± 1.20**	**0.986 ± 0.02**

## Data Availability

Human subject research has been done under an IRB of Case Western Reserve University (CWRU) and University Hospitals Health Systems (UHHS), Cleveland, OH. CT calcium score images were acquired at UHHS, de-identified, and made available via a material transfer agreement with University Hospitals Health Systems (UHHS). Any researcher who wants to gain access to this data must contact University Hospitals of Cleveland.
